# Characterization of Muffins Reformulated with Chia and Lyophilized Peach Powder in Terms of Some Technological and Sensory Aspects

**DOI:** 10.17113/ftb.61.03.23.7843

**Published:** 2023-09

**Authors:** Dasha Mihaylova, Aneta Popova, Zhivka Goranova, Pavlina Doykina, Bogdan Goranov

**Affiliations:** 1 Department of Biotechnology, University of Food Technologies, 26 Maritsa Blvd., 4002 Plovdiv, Bulgaria; 2 Department of Catering and Nutrition, University of Food Technologies, 26 Maritsa Blvd., 4002 Plovdiv, Bulgaria; 3 Institute of Food Preservation and Quality, Agricultural Academy, 154 Vasil Aprilov str., 4002 Plovdiv, Bulgaria; 4 Department of Microbiology, University of Food Technologies, 26 Maritsa Blvd., 4002 Plovdiv, Bulgaria

**Keywords:** cake, healthy alternative, fruit ingredient, *Prunus* spp., baked goods, technological factors, sensory evaluation

## Abstract

**Research background:**

There is an increasing interest in foods with added nutritional value. This study presents the opportunity for the reformulation of muffins using chia seeds and lyophilized peach powder in view of the emerging societal challenges regarding unhealthy eating patterns and food intolerances.

**Experimental approach:**

Two new formulations were developed to eliminate the use of eggs and alter the flour content and type. Physical characteristics, texture analysis, water activity, microbial load, antioxidant potential and sensory profile aided in the evaluation of the newly developed products.

**Results and conclusions:**

The results indicate an inversely proportional relationship between the relative mass of the dough and physical parameters (density, volume and height) of all muffin formulations. The modification of the original recipe compared to the control sample led to decreased baking losses, increased total phenolic content, as well as enhanced nutritional value in terms of fibre content. The addition of chia seeds and peach powder led to positive sensory changes. The alteration of the original recipe resulted in significant effect on the colour, making the muffins darker and less yellow than the control sample. In terms of texture characteristics, the new formulations had a profile close to the control.

**Novelty and scientific contribution:**

Recipe reformulation without deteriorating quality attributes is important for the food technology field. Reformulation should comply with the new expectations of the modern consumer. The study targets an approach where new products with enhanced functional characteristics are presented.

## INTRODUCTION

Food habits can significantly aid in maintaining good health and minimizing the manifestation of non-communicative diseases (*1*). The western diet is known for its high content of saturated fat, refined sugar and flour, low quantities of minerals, vitamins and antioxidants, which contributes to negative changes in the daily diet, decreased physical activity, gaining weight and poor health in general (*2*). For these reasons, recommendations of better and sustainable food choices are gaining popularity (*3*).

Baked goods like cookies, muffins, biscuits, cakes and waffles, among others, are desirable and preferred eating options due to their pleasant taste and market availability (*4*). If we have to evaluate their nutritional profile though, they are usually high in sugar, fat and energy and fall into the red zone of the traffic light food labelling system (*5*). Therefore, reformulating baked goods, in terms of a better nutritional profile, is a good option, especially considering the continuously rising level of overweight and obesity among children, adolescents and adults (*6*). What is more, introducing health-promoting ingredients such as antioxidants, fibre, minerals, and vitamins can positively alter these food formulations, promoting healthier eating habits, and long-term wellbeing of individuals (*7*, *8*).

The chia seed has a high nutritional potential due to its protein and fibre content, as well as a source of omega-3 and 6 fatty acids (*9*). Chia is also reported to aid the cardiovascular health (*10*), have antioxidant properties (*11*), exhibit anti-inflammatory properties (*12*), as well as better control of the lipid metabolism (*13*). Apart from that, whole grain consumption has been reported to contribute to a reduced risk of diseases like cardiovascular, diabetes, and some types of cancer (*14*). Einkorn flour has been successfully used in the preparation of dietetic dough (*15*). Additionally, fruits are very well-known for their beneficial properties (*16*), but are still limited to their consumption as fresh or dried. Furthermore, fruit-based products are usually associated with dairy (*17*). Genus *Prunus* representatives (peach, apricot, cherry, sour cherry, plum) are highly cherished for their sensory attributes and health-promoting properties (*18*). Numerous cultivars are gaining popularity due to their distinct profile, *i.e.* beneficial compounds, external properties and ripening period, among others. Peach ’Evmolpiya’ is a native Bulgarian variety, created by interspecific hybridization with the participation of *Prunus persica,* and *Prunus davidiana,* which ripens late in the summer season.

Nowadays, many studies focus on the effort to include ingredients with beneficial effects on human health that arise from the need to reformulate preferred but unhealthy foods. Muffins appear as a trending research area since scientific papers on their reformulation include the use of olive pomace flour (*19*), a kimchi by‐product (*20*), wheatgrass powder (*21*), sunflower flour (*22*) and green banana flour (*23*).

The main goal of this study is to determine the physical characteristics, texture profile, water activity, microbial load, antioxidant potential and sensory profile of reformulated muffins in order to enable the development of healthier products in terms of their nutritional value and quality characteristics.

## MATERIALS AND METHODS

### Materials

Fresh peach samples of the ’Evmolpiya’ variety were provided from the Fruit Growing Institute, Plovdiv, Bulgaria. The samples were lyophilized (vertical freeze dryer BK-FD12S; Biobase Biodustry Co., Ltd., Shandong, PR China) under the pressure of 3.5 MPa at −55 °C and powdered with Tefal GT110838 grinder (Rumilly, France) at 180 W for 30 s. The other products needed for the preparations were purchased from several stores in Plovdiv, Bulgaria, *i.e.* a local Lidl store (sugar, wheat flour, fresh eggs), a dm (organic chia seeds), and Balev bio (organic einkorn flour, organic baking powder). Wheat flour was produced and packaged by Good mills Jsc., Sofia, Bulgaria. The fresh eggs were produced and packaged by Medkovets Ltd., Gurmazovo village, Sofia province, Bulgaria. Sugar was produced and packaged by Zaharni zavodi, Gorna Oriyahovitsa, Bulgaria. Einkorn flour was produced and packaged by Ekosem Ltd, Stambolov village, Bulgaria. Chia seeds are imported from Peru (packaged by Internetkafe Ltd, Sofia, Bulgaria).

### Preparation of muffin formulations

**Fig. S1:**
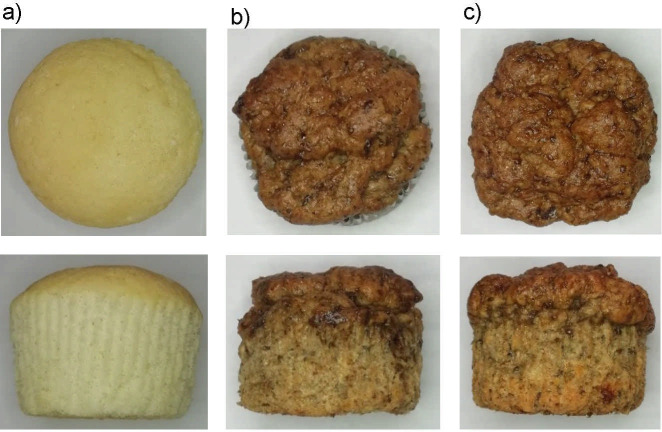
Muffin formulations: a) control sample, b) F1, and c) F2. F1 and F2=formulations containing sugar, water, baking powder, chia seeds, einkorn flour at 30 and 27 %, and peach powder at 3 and 6 %, respectively

The muffins (Fig. S1) were prepared using one-bowl baking method under laboratory conditions at the Institute of Food Preservation and Quality, Agricultural Academy, Plovdiv, Bulgaria. Table S1 provides information about the mass fraction of the ingredients used to prepare the formulations.

The control sample was prepared by beating the eggs and the sugar with a Philips mixer (HR 3745/00; Amsterdam, the Netherlands) at 450 W for 3 min until a fluffy light yellow mass was obtained, then adding the wheat flour and homogenizing again. The dough was dosed into muffin tins and baked in a preheated electric oven at 180 °C for 30 min. The amount of eggs in the newly developed recipes (formulations F1 and F2) was completely replaced by chia gel. Before preparing the muffins, chia seeds were ground in a Tefal grinder (model GT110838) at 180 W for 30 s. Chia gel was prepared by hydrating the chia powder in a *V*(chia):*V*(water)=1:9 for 30 min. All dry ingredients (sugar, flour, peach powder and baking powder) were previously mixed, and then mixed with the other ingredients (eggs or chia gel and water) for an additional minute. The dough (55 g) was dosed into muffin tins and baked in a preheated electric oven at 180 °C for 45 min. The baked muffins were cooled for 30 min. The samples were stored under standard conditions (temperature of (20±2) °С and relative humidity ≤75 %).

### Physical characteristics of the muffins

The height and volume of the muffins were measured using a digital calliper; the mass and baking losses were measured on an electronic scale. The baking loss was calculated by the following equation:

*m*(baking loss)=[(*m*(batter)–*m*(muffin))/*m*(batter)]·100 /1/

### Ash and moisture content

Ash mass fraction was determined by burning the weighed mass of sample in a muffle furnace according to AOAC 945.46 (*24*). Total moisture mass fraction of the samples was determined according to the procedure described in AACC method 44-15.02 (*25*).

### Nutritional data

The nutritional data were determined by the calculation method. The nutritional value of the finished products was calculated per 100 g based on specifications obtained from suppliers of each of the ingredients (sugar, flour, eggs and chia seeds). The nutritional value of the ’Evmolpiya’ peach was based on previous research (*26*).

### Colour

The CIELAB colour of the crust and crumb was analyzed with the use of PCE-CSM 2 (PCE-CSM instruments, Berlin, Germany) with a measuring aperture of 8 mm. The assessed parameters included lightness (*L*, ranging from 0 to 100), red-green opponent colours (representing *a*), blue-yellow opponent colours (representing *b*), chroma (*C*), and hue angle (*h*). The total colour difference (∆*E*) was calculated according to the CIE76 colour difference equation:


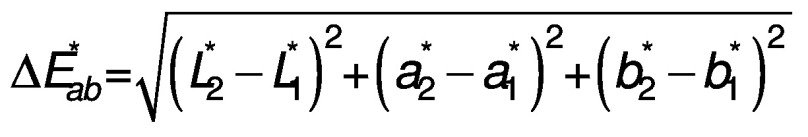
 /2/

where values for L*, a* and b* correspond to the CIELAB colour measurements.

### Browning index

Colour changes due to browning reaction were assessed using a colorimeter (L*, a*, b*) and browning index (BI) was measured following the equation:

BI=[100·(x–0.31)]/0.172 /3/

where x=(*a*+1.75·*L*)/(5.645·*L*+*a*–0.3012·*b*).

Values for L*, a* and b* correspond to the CIELAB colour measurements.

### Texture profile analysis

Texture profile analysis was performed by texture analyzer (TA.XT-2i Texture Analyzer Stable Microsystems, Godalming, Surrey, UK) with 0.15 N load cell following the AACC method 74-09 (*27*). The texture parameters (firmness, gumminess, chewiness, springiness and cohesiveness) were determined in a texture profile analysis mode. Texture analyzer test samples were prepared by cutting a cube from the middle of the muffins (2 cm×2 cm×2 cm), and tested with cylindrical probe of 36 mm, compression 60 %, pre-test, test and post-test speed of 2.0 mm/s and trigger force 5 g.

### Determination of total phenolic and total flavonoid content

The total phenolic content (TPC) was analyzed following a modified method of Kujala *et al.* (*28*). The TPC was expressed in mg gallic acid equivalents (GAE) per g of dry mass. The linear range for gallic acid standard was 5-100 mg/L (R^2^=0.9965). The total flavonoid content (TFC) was evaluated according to the method described by Kivrak *et al.* (*29*). Quercetin was used as a standard in the linear range of 5-80 μg/mL (R^2^=0.9972) and the results were expressed in μg quercetin equivalents (QE) per g of dry mass.

### Determination of antioxidant activity

The ability of the extracts to donate an electron and scavenge 2,2-diphenil-1-picrylhydrazyl (DPPH) radical was determined by the slightly modified method of Brand-Williams *et al.* (*30*) as described by Mihaylova *et al.* (*31*). The radical scavenging activity of the extracts against 2,2´-azino-bis(3-ethylbenzothiazoline-6-sulfonic acid) (ABTS^•+^) was estimated according to Re *et al.* (*32*). The Fe^3+^ reducing antioxidant power (FRAP) assay was carried out according to a slightly modified procedure of Benzie and Strain (*33*). Prior to use, the freshly prepared FRAP reagent was warmed to 37 °C. The extracts were allowed to react with 2.85 mL of the FRAP reagent for 4 min at 37 °C, and the absorbance was measured at 593 nm. The Cu^2+^ reducing antioxidant capacity (CUPRAC) assay, which enables the total antioxidant capacity measurements of hydrophilic as well as hydrophobic samples. was carried out according to the procedure of Apak *et al.* (*34*).

### Water activity and microbial count

The water activity and microbial load of the samples were measured as described by Mihaylova *et al.* (*35*).

### Microscopic imaging

The photographs of the pores of the muffins were obtained *via* a USB Digital pocket microscope MX200-B (T TAKMLY, Shenzhen Huishixin Technology Co Ltd, Shenzhen, PR China) with 1000× LED magnification endoscope camera and a focus range of 1-9 mm. ImageJ software (*36*) was used to calculate the size of the pores.

### Sensory evaluation

Sensory analysis was performed at the University of Food Technologies, Plovdiv, Bulgaria. The evaluation followed the description of Mihaylova *et al.* (*37*). Attributes of the evaluation were appearance (*N*=8), aroma (*N*=5), taste (*N*=6), mouthfeel (*N*=8) and aftertaste (*N*=7).

### Statistical analysis

Data were analyzed using MS Excel software (*38*). All assays were performed in at least three repetitions. Results were presented as mean value±standard deviation (S.D.). Relevant statistical analyses of the data were performed using one-way ANOVA and a Tukey-Kramer *post hoc* test (α=0.05), as described by Assaad *et al.* (*39*).

## RESULTS AND DISCUSSION

In order to accurately compare the newly developed formulations, several parameters were considered, *i.e.* moisture and ash content, nutritional data, crust and crumb colour, texture analysis, pore distribution, TPC, TFC, antioxidant activity, browning index, water activity and sensory evaluation.

Table 1 shows the physical properties and nutritional data of the muffin formulations. The control sample was characterised with the highest volume and height, and with the addition of chia seeds and peach powder these parameters decreased. Other authors have found similar results with the addition of ingredients like grape pomace (*40*), cocoa fibre (*41*) and kimchi powder (*20*).

**Table 1:** Physical characteristics, ash, moisture, water activity, microbial load and nutritional data of muffins

Sample	Control			F1			F2		
*h*/cm	(7.2±1.0)^a^			(5.3±0.1)^b^			(5.4±0.3)^b^		
*m*/g	(36.3±5.1)^b^			(61.1±3.9)^a^			(51.6±7.8)^a^		
Loss rate/%	(11.6±1.6)^a^			(3.2±1.4)^b^			(3.63±0.9^b^		
*V*/cm^3^	(21.2±0.3)^a^			(9.1±0.8)^b^			(10.5±1.5)^b^		
*w*(ash)/%	(1.1±0.2)^a^			(1.5±0.3)^a^			(1.00±0.07)^a^		
*w*(moisture)/%	(20.2±0.6)^c^			(35.2±1.3)^a^			(30.6±2.8)^b^		
*a*_w_	(0.760±0.001)^c^			(0.89±0.01)^a^			(0.84±0.01)^b^		
* t*(testing)/h	0	48	96	0	48	96	0	48	96
*N*(AMM)/(CFU/g)	2·10^1^	3·10^2^	3.5·10^2^	4·10^1^	10^2^	3.2·10^2^	3·10^1^	1.5·10^2^	3·10^2^
*N*(YM)/(CFU/g)	2.2·10^2^	<10	<10	10^3^	<10	<10	1.5·10^3^	<10	<10
*w*(protein)/(g/100 g)	8.79			4.04			3.71		
*w*(carbohydrate)/(g/100 g)	49.32			23.47			23.27		
*w*(sugar)/(g/100 g)	27.36			4.90			5.04		
*w*(fibre)/(g/100 g)	0			4.25			4.24		
*w*(fat)/(g/100 g)	5.7			1.82			1.82		
*w*(saturated fat)/(g/100 g)	1.48			0.13			0.13		
*E*/kcal	276.98			123.51			121.92		

Different letters in superscript in the same row indicate statistically significant differences (p<0.05), according to one-way ANOVA and the Tukey’s test. F1 and F2=formulations containing sugar, water, baking powder, chia seeds, einkorn flour at 30 and 27 %, and peach powder at 3 and 6 %, respectively, AMM=aerobic mesophilic microorganisms, YM=yeasts and mould, CFU=colony forming units

The mass of the reformulated muffins increased and the baking loss decreased. These findings correspond well to other papers concerning newly developed muffin formulations (*20*, *23*). Since the final volume is particularly important for bakery products, it has to be noted that other researchers have documented similar results, which are most likely due to the presence of dietary fibre and the dilution of gluten (*42*).

Information about the carbohydrate, protein and fat content is introduced in Table 1. The new formulations are characterised with reduced sugar and carbohydrate mass fraction compared to the control sample. Between the two formulations (F1 and F2), it can be seen that they bear very similar nutritional values. Muffins, in general, are characterised with an increased fat and sugar amount, thus reformulation in this direction is important not only because of the rising obesity worldwide, but also because products with a target nutritional profile and consumer acceptability may aid in reducing obesity rates (*43*). Following the Regulation (EC) No 1924/2006 (*44*) of the European Parliament and of the Council of 20 December 2006 on nutrition and health claims made on foods, the newly developed formulations are sources of fibre since they provide 4.24 g/100 g in formulation F2 and 4.25 g/100 g in formulation F1. Expectedly, the dietary fibre mass fraction increased with the incorporation of chia seeds.

Determining the moisture and ash mass fraction as well as the nutritional data of the end product is important. The moisture mass fraction of the control and the newly formulated ones varied from (20.23±0.61) to (35.15±1.28) %. The lowest values were established in the control sample, indicating that the absence of eggs and their substitution with chia seeds in formulations F1 and F2 led to a more moist product, which is rather untypical for baked samples. Other papers document 21.71 to 23.90 % moisture in muffins formulated with kimchi by-product powder (*20*), which is similar to the control sample of the current study. The ash mass fraction was in the range from 1 to 1.53 %. The highest values were established in formulation F1. Considering the ash mass fraction, other papers show similar values (*20*).

The *a*_w_ of the formulations was evaluated and the results varied from 0.760±0.001 in the control sample to 0.89±0.01 in F1, which had the highest moisture content (Table 1). These results are lower than the ones documented by Struck *et al.* (*45*) about fibre-enriched sugar-reduced muffins where the water activity ranged from 0.85 to 0.98. However, F1 had more water available for reactions and microorganisms to use. The *a*_w_ recorded for F2 was 0.8±0.01. The results correspond well to the established moisture content in the muffin formulations. The recorded microbial load at 0, 48 and 96 h of storage showed that the muffin formulations are safe for consumption. The percentage of added peach powder did not significantly influence the total count of yeast, mould and aerobic mesophilic microorganisms (AMM). The established *a*_w_ values correspond well to the AMM count.

**Fig. 1:**
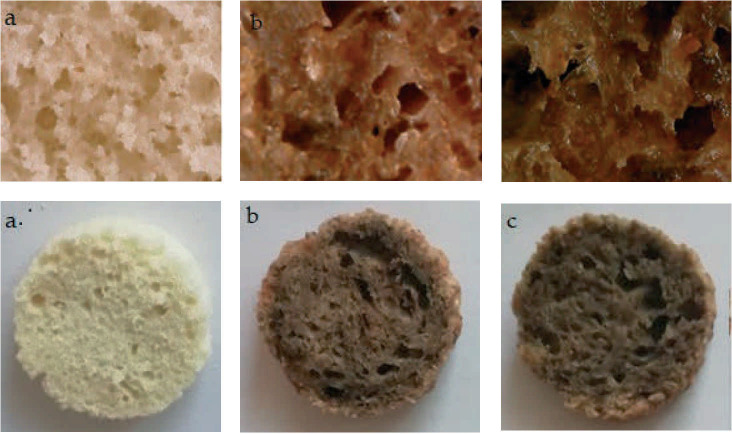
Photographs and micrographs of muffin pores: a) control sample, b) sample F1, and c) sample F2. F1 and F2=formulations containing sugar, water, baking powder, chia seeds, einkorn flour at 30 and 27 %, and peach powder at 3 and 6 %, respectively

Fig. 1 shows cross sections of horizontally cut muffins as well as micrographs of the pores of all studied samples. It can be easily seen that the inclusion of chia seeds and peach powder has led to a decreased air cell formation.

The control sample contained small and uniform air pockets, while F1 and F2 had irregular ones, and tunnel-like areas. This describes the same trend as observed by other researchers who studied reformulated muffins (*20*). Control sample had the smallest pores ((1.4±0.6) mm), while F1 and F2 had pores in the range from (3.19±1.08) to (3.80±0.63) mm.

Texture attributes represented by hardness, gumminess, chewiness, springiness and cohesiveness, as well as crust and crumb colour of the formulations described with L*, a*, b*, *C* and *h* values, are presented in Table 2. Both texture and colour characteristics can affect the perception of a food product and its overall acceptance.

**Table 2:** Colour characteristics (L*, a*, b*, *C* and *h*), browning index, and texture attributes of the muffins

Sample	L*	a*	b*	*C*	*h*	BI
Crust						
Control	(74.8±1.8)^a^	(9.5±0.9)^d^	(32.3±0.6)^b^	(33.7±0.7)^b^	(73.7±1.4)^a^	(13.2±1.2)^c^
F1	(29.5±3.6)^a^	(13.4±1.3)^e^	(18.7±0.6)^d^	(23.1±0.9)^d^	(54.5±2.8)^b^	(36.9±3.7)^c^
F2	(34.5±2.6)^b^	(15.3±0.8)^e^	(19.7±1.6)^d^	(25.0±1.7)^c^	(52.1±1.1)^a^	(35.2±1.9)^b^
Crumb						
Control	(75.7±1.2)^b^	(1.1±0.2)^e^	(17.6±0.8)^c^	(17.6±0.8)^c^	(86.3±0.5)^a^	(3.3±0.2)^d^
F1	(39.3±1.9)^b^	(8.4±0.2)^e^	(17.1±0.9)^d^	(19.3±0.9)^c^	(54.3±1.1)^a^	(19.4±1.0)^c^
F2	(40.9±1.9)^b^	(8.1±0.3)^d^	(19.1±1.5)^c^	(20.8±1.3)^c^	(66.9±1.7)^a^	(18.4±1.0)^c^
Texture profile						
	Hardness/N Cycle 1	Gumminess/N	Adhesiveness/(N/mm^2^)	Springiness/mm	Cohesiveness	Chewiness/J
	Cycle 2					
Control	10537±16	(6427±416)^c^	(0.6±0.02)^c^	(3.5±0.1)^b^	(0.6±0.03)^c^	(22573±1882)^c^
	8235±14					
F1	7267±25	(23692±1135)^b^	(5.2±0.2)^b^	(2.7±0.3)^c^	(3.3±0.2)^b^	(62932±5431)^b^
	6486±30					
F2	12325±20	(53334±3844)^a^	(6.8±0.1)^a^	(4.2±0.3)^a^	(4.2±0.3)^a^	(164156±79357)^a^
	10347±30					

Different letters in superscript in the same column indicate statistically significant differences (p<0.05), according to one-way ANOVA and the Tukey’s test. F1 and F2=formulations containing sugar, water, baking powder, chia seeds, einkorn flour at 30 and 27 %, and peach powder at 3 and 6 %, respectively, BI=browning index

The results reveal that the control sample is harder than F1 but less hard than F2. This tendency is similar to the one reported by Najjar *et al.* (*46*) concerning the texture profile of cookies formulated with date seed powder. The absence of eggs resulted in increased values in the adhesiveness and cohesiveness parameters. Lower cohesiveness is associated with increased crumbliness due to greater hardness (*40*). This thesis is further supported by the sensory evaluation of the muffin formulations presented later in this research. Moreover, in sample F1, the springiness was the lowest (2.66 mm). Springiness is an important mechanical feature associated with an elastic and fresh aerated product (*47*). The adhesiveness and cohesiveness were highly influenced by the recipe alteration. Egg white protein aids in the air and volume formation. Its absence leads to products with smaller volume, which is visible in the formulations with chia seeds. This is most likely because of the increased density and reduced number of air pockets. Chewiness reflects the difficulty needed to chew food and form bolus prior to swallowing (*48*). The formulated samples require additional force to chew them and there is a proportional reference between the mass fraction of added peach powder and the chewiness values.

The newly developed formulations appeared darker than the control. The difference in colour is due to the initial colour of the ingredients. Moreover, the presence of chia seeds and einkorn flour resulted in lighter inner part, with 15 % higher L* and *h* values. The crust of formulations F1 and F2 appeared darker, with higher a* and lower b* values, than of control sample. The F1 and F2 samples may appear the same to some consumers as they lean to the brown shade with weak hints of the yellow and red. The two new formulations had similarities in the colour perception, while the control sample was significantly different in its crust and crumb colour parameters. The calculated ∆*E* of the crust of F1 (47.46) and F2 (42.60) shows that the human eye perceives a difference between samples, but the newly developed samples are more similar in colour than different. The colour parameters are difficult to compare to other products because of the differences between recipe designs.

The values of the browning index were significantly different compared to the control sample and relatively similar for the newly formulated muffins (Table 2). The same trend was followed for both crumb and crust. The current results support the thesis of Shevkani and Singh (*49*) who state that the browning index very much depends on the initial ingredient colour of the product.

The TPC, expressed in GAE on dry mass basis, of the control sample was (0.06±0.00) mg/g. A fourfold increase was observed for the two new formulations, resulting in (0.25±0.01) (F1) and (0.32±0.00) mg/g (F2). Similarly, an increase in TPC was observed in muffins prepared with wheatgrass powder, with a 1.4-fold increase compared to the control sample (*21*). The TFC value of the control sample, expressed as QE on a dry mass basis, was zero, which is consistent with its ingredients. The F1 and F2, on the other hand, had (18.7±0.8) and (31.2±0.4) μg/g, respectively. Similar results were reported for muffins fortified with capsicum pomace powder (*50*).

**Fig. 2:**
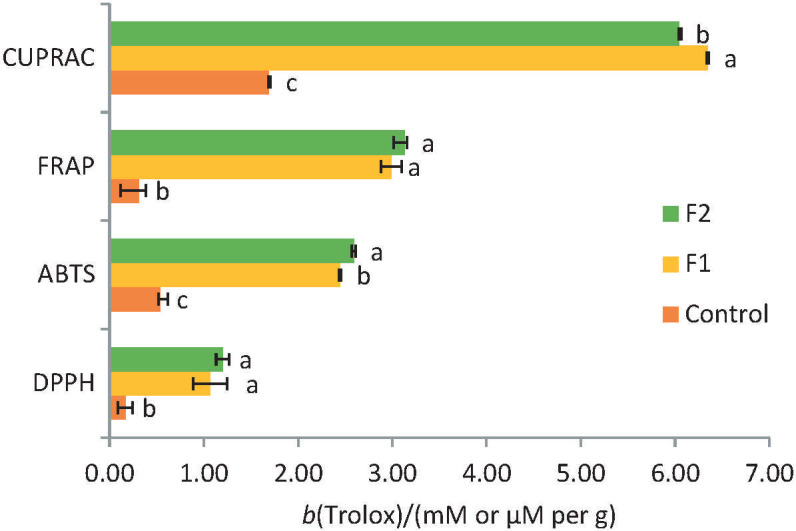
Antioxidant activity of muffin formulations measured on dry mass basis by ABTS (mM/g), DPPH (µM/g), FRAP (µM/g) and CUPRAC (µM/g) assays. Different letters in the same assay indicate statistically significant differences (p<0.05), according to one-way ANOVA and the Tukey’s test. F1 and F2=formulations containing sugar, water, baking powder, chia seeds, einkorn flour at 30 and 27 %, and peach powder at 3 and 6 %, respectively

The addition of peach powder and chia seeds, as well as flour substitution led to a significant increase in the antioxidant activity measured by four contemporary methods (Fig. 2). Other authors also found the increase in their samples (*21*, *22*) based on their ingredients *i.e.* sunflower flour and wheatgrass powder.

The highest values were measured by the ABTS assay. Formulations F1 and F2 had similar results, which is in accordance with their TPC. Some authors state that a higher natural antioxidant content can have a positive influence of the shelf life of baked goods (*51*). Here, the addition of chia seeds and peach powder increased the overall bioactive profile of the muffins. Similarly, studies have shown that levels of FRAP increase 2.9-fold in in muffins prepared with sunflower flour (*22*).

The evaluation of sensory properties of the muffins is presented in Table 3. In terms of appearance, the muffins had distinct differences. The colour of the control sample was yellowish, while the two other formulations were described as golden brown. All samples were considered uniform in colour. Formulations F1 and F2 left a sticky feeling on the fingers and had an uneven surface top. The control sample, on the other hand, was marked with an even top surface, but very crumbly when pulled apart.

**Table 3:** Sensory evaluation of muffin formulations

Attribute/sample	Control	F1	F2
Appearance			
yellow colour	(12.3±1.9)^a^	(1.0±0.7)^b^	(0.9±0.6)^b^
golden brown colour	(0.0±0.0)^b^	(12.7±0.9)^a^	(13.3±1.2)^a^
variation of pore size	(3.5±1.6)^b^	(11.9±1.2)^a^	(12.6±1.2)^a^
sticky to touch	(4.7±1.0)^b^	(13.5±0.8)^a^	(13.8±0.9)^a^
dry to touch	(10.6±1.7)^a^	(1.7±0.8)^b^	(2.0±0.8)^b^
crumbly when pulled apart	(12.4±1.3)^a^	(2.6±1.4)^b^	(3.7±1.3)^b^
uneven top surface	(1.7±0.8)^b^	(10.6±1.6)^a^	(11.2±1.3)^a^
uniformity of colour	(13.4±1.4)^a^	(12.4±1.1)^a^	(12.2±1.0)^a^
Aroma			
sweet	(9.7±1.9)^b^	(13.4±1.0^5^	(13.1±1.2)^a^
egg-like	(9.3±0.7)^a^	(0.3±0.48)^b^	(0.3±0.5)^b^
pumpkin-like	(0.0±0.0)^c^	(9.8±1.3)^b^	(11.4±1.8)^a^
fruity	(0.0±0.0)^b^	(4.7±2.0)^a^	(5.8±1.6)^a^
caramel-like	(0.9±0.6)^b^	(3.9±1.7)^a^	(3.8±1.8)^a^
Taste			
sweet	(14.2±0.8)^a^	(12.2±0.9)^b^	(12.3±1.0)^b^
egg-like	(9.3±1.5)^a^	(0.0±0.0)^b^	(0.00±0.00)^b^
fruity	(2.3±1.2)^b^	(4.7±1.9)^a^	(5.6±1.0)^a^
flour-like	(6.1±1.2)^a^	(3.5±1.3)^b^	(3.5±1.6)^b^
insipid (flavourless)	(0.6±0.5)^b^	(0.2±0.4)^b^	(1.6±1.0)^a^
bitter	(0.8±0.6)^a^	(1.9±1.6)^a^	(1.6±1.0)^a^
Mouthfeel			
crunchy	(1.4±0.5)^b^	(6.0±0.8)^a^	(5.1±1.4)^a^
tooth packing	(5.4±1.3)^a^	(6.3±1.6)^a^	(5.7±1.5)^a^
tongue film-forming	(6.1±1.2)^a^	(4.8±1.3)^a^	(5.0±1.2)^a^
salivating	(7.1±1.2)^b^	(8.9±1.2)^a^	(8.9±1.0)^a^
dry	(8.9±1.0)^a^	(3.2±1.3)^b^	(3.3±1.9)^b^
sticky	(5.4±1.4)^b^	(8.9±0.7)^a^	(8.7±0.9)^a^
difficult to bite	(2.7±1.5)^a^	(2.2±0.8)^a^	(2.7±1.2)^a^
soft	(8.5±1.6)^a^	(7.9±1.1)^a^	(7.6±1.4)^a^
Aftertaste			
sweet	(12.2±1.2)^a^	(11.1±1.2)^a^	(12.3±1.2)^a^
egg-like	(5.1±1.9)^a^	(0.0±0.0)^b^	(0.0±0.0)^b^
dry	(3.3±1.5)^a^	(1.7±0.8)^b^	(2.0±0.8)^b^
salivating	(5.1±1.2)^a^	(3.5±1.1)^b^	(3.1±1.5)^b^
flour-like	(2.1±1.0)^a^	(1.7±0.9)^a^	(2.5±1.1)^a^
fruity	(0.0±0.0)^b^	(3.5±1.1)^a^	(3.7±1.5)^a^
bitter	(1.3±0.7)^a^	(1.5±0.8)^a^	(2.0±1.0)^a^

Different letters in the same row indicate statistically significant differences (p<0.05) according to one-way ANOVA and the Tukey’s test. F1 and F2=formulations containing sugar, water, baking powder, chia seeds, einkorn flour at 30 and 27 %, and peach powder at 3 and 6 %, respectively

The inside of the muffins had different pore size and distribution. The control sample had the smallest pores, while F1 and F2 had bigger, unevenly distributed pores. This is in accordance with the findings discussed in Fig. 1. The aroma was predominantly egg-like and sweet for the control sample, and pumpkin-like and sweet for F1 and F2. When tasting the samples, the panellists marked the sweet taste as predominant. Formulations F1 and F2 were also described as pleasantly crunchy, but forming a bolus in the mouth. The control sample on the other hand was dry and falling apart. The aftertaste of all muffin formulations was the sweet one. Since all formulations were well accepted by the panel, it can be concluded that the presence of 3 and 6 % of peach powder is acceptable. The absence of eggs, even though it led to differences in the surface top, was also accepted and the texture of the chia seeds in the ready product was not unpleasant.

The Pearson’s correlation coefficients between water activity, moisture mass fraction, antioxidant activity, TPC, TFC, browning index and CIELAB crust colour characteristics of muffin formulations are plotted in Table 4. All variables have a positive correlation, and the samples were considered to have a very high correlation, with a coefficient ≥0.75. A high degree of correlation is marked when 0.74≤R^2^≥0.50, while moderate degree of correlation is when the value lies between ±0.30 and ±0.49.

**Table 4:** Pearson’s correlation coefficients (R^2^) between water activity, moisture content, antioxidant activity (DPPH, ABTS, FRAP and CUPRAC assays), total phenolic content, total flavonoid content, browning index and CIELAB crust colour characteristics of muffin formulations

Control sample	BI	L*	a*	b*	*C*	*h*	WA	MC	TPC	TFC	DPPH	ABTS	FRAP	CUPRAC
BI	1	0.997	0.998	0.639	0.839	0.967	0.479	0.464	0.312	0.943	0.446	0.955	0.621	0.097
L*	0.997	1	1	0.581	0.794	0.985	0.538	0.523	0.368	0.913	0.388	0.928	0.677	0.134
a*	0.998	1	1	0.596	0.806	0.981	0.523	0.509	0.354	0.921	0.402	0.935	0.664	0.124
b*	0.639	0.581	0.596	1	0.947	0.458	0.014	0.011	0.003	0.845	0.963	0.825	0.067	0.104
*C*	0.839	0.794	0.806	0.947	1	0.686	0.119	0.11	0.032	0.971	0.828	0.961	0.226	0.009
*h*	0.967	0.985	0.981	0.458	0.686	1	0.659	0.645	0.49	0.831	0.272	0.851	0.786	0.229
WA	0.479	0.538	0.523	0.014	0.119	0.659	1	1	0.971	0.25	0.006	0.274	0.98	0.812
MC	0.464	0.523	0.509	0.011	0.11	0.645	1	1	0.976	0.238	0.008	0.261	0.975	0.823
TPC	0.312	0.368	0.354	0.003	0.032	0.49	0.971	0.976	1	0.119	0.06	0.137	0.904	0.925
TFC	0.943	0.913	0.921	0.845	0.971	0.831	0.25	0.238	0.119	1	0.682	0.999	0.383	0.006
DPPH	0.446	0.388	0.402	0.963	0.828	0.272	0.006	0.008	0.06	0.682	1	0.656	0.005	0.25
ABTS	0.955	0.928	0.935	0.825	0.961	0.851	0.274	0.261	0.137	0.999	0.656	1	0.41	0.01
FRAP	0.621	0.677	0.664	0.067	0.226	0.786	0.98	0.975	0.904	0.383	0.005	0.41	1	0.689
CUPRAC	0.097	0.134	0.124	0.104	0.009	0.229	0.812	0.823	0.925	0.006	0.25	0.01	0.689	1
F1	**BI**	L*	a*	b*	***c***	***h***	**a_w_**	**m**	**TPC**	**TFC**	**DPPH**	**ABTS**	**FRAP**	**CUPRAC**
BI	1	0.451	0.967	0.739	0.997	0.93	0.002	0.727	0.843	0.922	0.033	0.363	0.216	0.784
L*	0.451	1	0.277	0.913	0.394	0.204	0.589	0.035	0.104	0.191	0.724	0.992	0.118	0.882
a*	0.967	0.277	1	0.567	0.985	0.993	0.02	0.871	0.95	0.99	0	0.201	0.381	0.619
b*	0.739	0.913	0.567	1	0.687	0.482	0.297	0.218	0.345	0.466	0.434	0.856	0.003	0.997
*C*	0.997	0.394	0.985	0.687	1	0.956	0	0.777	0.882	0.95	0.016	0.308	0.265	0.735
*h*	0.93	0.204	0.993	0.482	0.956	1	0.051	0.923	0.981	1	0.007	0.137	0.466	0.534
WA	0.002	0.589	0.02	0.297	0	0.051	1	0.238	0.129	0.058	0.98	0.675	0.75	0.25
MC	0.727	0.035	0.871	0.218	0.777	0.923	0.238	1	0.98	0.931	0.128	0.009	0.738	0.262
TPC	0.843	0.104	0.95	0.345	0.882	0.981	0.129	0.98	1	0.985	0.049	0.056	0.605	0.395
TFC	0.922	0.191	0.99	0.466	0.95	1	0.058	0.931	0.985	1	0.01	0.126	0.482	0.518
DPPH	0.033	0.724	0	0.434	0.016	0.007	0.98	0.128	0.049	0.01	1	0.801	0.617	0.383
ABTS	0.363	0.992	0.201	0.856	0.308	0.137	0.675	0.009	0.056	0.126	0.801	1	0.182	0.818
FRAP	0.216	0.118	0.381	0.003	0.265	0.466	0.75	0.738	0.605	0.482	0.617	0.182	1	0
CUPRAC	0.784	0.882	0.619	0.997	0.735	0.534	0.25	0.262	0.395	0.518	0.383	0.818	0	1
F2	BI	L*	a*	b*	*c*	*h*	a_w_	m	TPC	TFC	DPPH	ABTS	FRAP	CUPRAC
BI	1	0.554	0.247	0.002	0.032	0.277	0.498	0.616	0.049	0.531	0.217	0.179	0.948	0.468
L*	0.554	1	0.044	0.397	0.274	0.921	0.003	0.996	0.236	0.007	0.06	0.084	0.328	0.993
a*	0.247	0.044	1	0.794	0.889	0.227	0.932	0.022	0.915	0.914	0.999	0.993	0.464	0.086
b*	0.002	0.397	0.794	1	0.983	0.678	0.551	0.336	0.97	0.518	0.823	0.857	0.076	0.483
*C*	0.032	0.274	0.889	0.983	1	0.551	0.678	0.22	0.998	0.646	0.91	0.935	0.159	0.354
***h***	0.277	0.921	0.227	0.678	0.551	1	0.053	0.884	0.507	0.039	0.257	0.3	0.102	0.961
**WA**	0.498	0.003	0.932	0.551	0.678	0.053	1	0.013	0.718	0.999	0.913	0.885	0.72	0.001
**MC**	0.616	0.996	0.022	0.336	0.22	0.884	0.013	1	0.185	0.022	0.033	0.052	0.389	0.978
**TPC**	0.049	0.236	0.915	0.97	0.998	0.507	0.718	0.185	1	0.687	0.934	0.955	0.192	0.313
**TFC**	0.531	0.007	0.914	0.518	0.646	0.039	0.999	0.022	0.687	1	0.893	0.863	0.749	0
**DPPH**	0.217	0.06	0.999	0.823	0.91	0.257	0.913	0.033	0.934	0.893	1	0.998	0.429	0.107
**ABTS**	0.179	0.084	0.993	0.857	0.935	0.3	0.885	0.052	0.955	0.863	0.998	1	0.382	0.138
**FRAP**	0.948	0.328	0.464	0.076	0.159	0.102	0.72	0.389	0.192	0.749	0.429	0.382	1	0.25
**CUPRAC**	0.468	0.993	0.086	0.483	0.354	0.961	0.001	0.978	0.313	0	0.107	0.138	0.25	1

MC=moisture content, BI=browning index, *L, a, b, C, h*=CIELAB parameters, WA=water activity, TPC=total phenolic content, TFC=total flavonoid content, F1 and F2=formulations containing sugar, water, baking powder, chia seeds, einkorn flour at 30 and 27 %, and peach powder at 3 and 6 %, respectively

It can be seen that a very limited number of statistically independent variables, with a correlation coefficient equal to zero, exists in this study. The antioxidant activity, TPC and TFC are very commonly correlated in research papers (*52*). The R^2^ values indicate that the phenolic and flavonoid compounds are contributors to the antioxidant activity in all muffin formulations at different levels. Good correlation coefficients were established considering the colour characteristics and the TPC, TFC and antioxidant activity. Other papers have also determined a correlation between colorimetric values and TPC and TFC (*53*).

## CONCLUSIONS

Muffins are generally very well accepted by consumers and are often chosen as a dessert or snack throughout the day. Faced with increased calorie intake and unhealthy eating habits, the consumer demands healthier alternatives of desired goods. Thus, the impact of the inclusion of peach powder, einkorn wheat, and chia seeds as egg substituent in muffins was studied. Reformulation led to a product with less energy, sugars and fat, as well as an increased fibre content and antioxidant activity, in line with the current trend for foods with enhanced functional properties. The newly developed products showed good results in terms of quality and based on the comparison of the studied parameters with the control sample. The colour of the newly developed muffin formulations was significantly different from the control sample, mostly due by the ingredients (flour type, chia seeds). The micrographs showed pores with increased size and the sensory evaluation pointed out a distinct stickiness in the formulated muffins. However, both new formulations were generally well-accepted by the panellists. The results of this study indicate that a potential for the formulations of muffins with chia seeds and lyophilized peach powder exists without deteriorating important quality attributes. Future reformulation could target the sugar (total substitution) and protein (increase) content of the muffins.

## Appendix

**Table S1:** Muffin formulations

Ingredient	Control	*w*(ingredient)/% F1	F2
Sugar	26	28	28
Wheat four	32	0	0
Einkorn flour	0	30	27
Eggs	42	0	0
Chia seeds	0	4	4
Peach powder	0	3	6
Water	0	34.8	34.8
Baking powder	0	0.2	0.2

F1 and F2=formulations containing sugar, water, baking powder, chia seeds, einkorn flour at 30 and 27 %, and peach powder at 3 and 6 %, respectively
